# Professional coaching versus vocal coaching: similarities and differences

**DOI:** 10.1590/2317-1782/20212021003

**Published:** 2022-02-23

**Authors:** Mara Behlau, Glaucya Madazio, Claudia Pacheco, Thays Vaiano, Flávia Badaró, Marisa Barbara

**Affiliations:** 1 Centro de Estudos da Voz – CEV, São Paulo (SP), Brasil.; 2 Universidade Federal de São Paulo – UNIFESP, São Paulo (SP), Brasil.; 3 Insper, São Paulo (SP), Brasil.

**Keywords:** Coaching, Training, Performance, Voice, Communication

## Abstract

**Purpose:**

This Brief Communication describes the professional coaching/coach counterpointed by the vocal coaching/coach. The aim is to introduce and explain these two coaching perspectives for a correct and specific use of the terms.

**Methods:**

Six undergraduate professors, speech language pathologists – SLP and/or professional coaches and/or vocal coaches met and shared their perceptions and experiences in professional or vocal coaching and in teaching coaching strategies to professionals working in the voice field. A chart was set encompassing the similarities and differences between the two attributions, both in terms of the intervention process and professional training.

**Results:**

Six fundamental aspects were identified to characterize the two coaching presentations, both professional and vocal, namely: credentials, performance, process, basic knowledge, partnership for results, and professional title.

**Conclusion:**

Professional coaching involved a structured process that requires certified training with individuals accredited by qualified associations aiming to facilitate positive changes in clients’ lives and improve for the understanding of how personal and professional skills are developed. Conversely, vocal coaching can be characterized as an unstructured function that does not require accredited training, executable by various health or vocal pedagogy professionals for improving voice and/or communicative performance of artistic or non-artistic individuals , sung or spoken voice users. Particularly regarding vocal coaching, it is suggested that the professional is identified throughout the academic training or basic professional performance, thus adding the term vocal coach as a qualifier.

## INTRODUCTION

The origin of the word coaching is not well defined, but it has been recorded since the 16th century, in medieval Hungarian, as *kocsi*, as a reference to the famous “Kocs coaches”, in a town in which these luxurious transportation modes were built. From *kocsi* to *coche*, in French, and *kotsche*, in German, the entry appears as coach later in England, in the first half of the 19th century, thus indicating a private teacher who guides a student through the learning process before submitting them to an exam. In the second half of that same century, both in England and in the United States of America (USA), the word coach was used specifically to refer to an instructor or sports coach in the scope of athletic^(1)^training practices. Therefore, the meaning of coach is associated with someone who monitors an individual in a sophisticated journey and assists in reaching a specific target. From the sports scenario, coaching was transferred to the world of organizations in the 1970s and 1980s, producing a huge degree of diversification in the 1990s, as well as becoming professionalized, later becoming a popular job in this century, consolidated as one of the fastest growing professions in the world^(2)^.

Thus, the words coaching and coach have been increasingly used both in lay texts and in the scientific literature, always maintaining its English form, with no equivalent in Portuguese. Coaching is related to a process, whereas coach is associated with the professional qualified for this specific activity. The differences are not clear between these procedures and other service modalities, as well as between the professionals responsible for it and other service renderers, such as teachers/professors, physicians, and sports coaches. Although coaching generally refers to a human development approach, it may also have contributed to understand the meaning of such process and position. Qualifiers are frequently added indicating the context in which the term is being used, such as professional, executive, career, systemic, life, sports, and vocal coaching/coach. The conductor of the process is called ‘coach’, whereas the person who hires the service is initially called ‘coachee’. However, more recently, the term client has been preferred since it is simpler to pronounce and easily to understand in Portuguese^(3)^. 

Many studies in the literature have addressed the professional coaching and its contributions in organizations, whose results could demonstrate the effects in productivity and executive performance, with variable levels of evidence^(4)^. Still, medical education is also presented as a training modality with high evidence supporting the use of this process to improve technical skills^(5)^.

In the Speech-Language pathology area, voice specialists have used both terms, coaching and vocal coach, more frequently in social media, thus being relevant to differentiate them. Literature addressing vocal coaching is very limited and there is only one specific paper^(6)^ that represents a summary of a singing teachers‘ panel, during the 10th Pan-European Voice Conference (PEVOC), in which three voice pedagogy areas are proposed: voice building, vocal coaching, and voice rehabilitation. In this panel, vocal coaching is presented as the capacity to transfer and establish skills that are directly related to stage or studio performance and artistic expression. The main requirement to perform the function of vocal coach is to have active or support professional experience in vocal performance or studio recording. Such a proposal is considered relevant in the area, although it has not been deeply explored yet. Recently, a tutorial presenting the use of coaching strategies in voice training and rehabilitation of individuals with behavioral dysphonia has allowed an overview of resources for this interaction modality that may be used both in the health area and in vocal pedagogy^(7)^.

This paper aimed to compile the main similarities and differences between professional coaching and vocal coaching, as well as to promote transparency between processes related to these two scenarios and to the professional positions developed by individuals who provide such attendance.

## METHODS

This paper results from a reflection of the authors, all post-graduation professors. Since the research does not use any personal data of the participating individuals, neither the approval of Ethics Committee nor Free, Prior and Informed Consent (FPIC) was required.

Six professors of a postgraduate program, training level, met to share their perceptions and experiences on professional coaching and vocal coaching processes, as well as on the performance of professional and vocal coaches. Six basic aspects were selected for characterizing both of the coaching categories, as follows: 1. Qualification: credential for professional practice and certification levels needed; 2. Performance: maintaining a professional approach to the client; 3. Work process: definition of objectives, contract, and duration of attendance; 4. Basic knowledge in the area demanded by the client: relevant training on the issues raised by the client; 5. Partnership for results: characterize the relationship between coach and client, pointing out the responsibility for the results, and 6. Professional title: identification of coach qualification. Box 1 introduces the results of the discussion that encompassed three one-hour meetings.

**Box 1:** Main similarities and differences between professional and vocal coaching and coach

**ASPECTS**	**PROFESSIONAL COACHING/COACH**	**VOCAL COACHING/COACH**
**EDUCATION**	Title in a Professional coaching School (accredited to ICF or other accredited institution); ICF accredits coaches in three levels: ACC, PCC an MCC; has a code of ethics and eight competencies to be followed^(3)^; professionals of the most varied academic formations aim to obtain a certification to act as professional coaches.	It does not require a title and anyone, without specific training, may be called a vocal coach; the market recognizes the worth of this specialty; there is no level of certification; the professional is expected to abide to the code of ethics of his/her profession and there is no description of the competencies; the vocal coach function is carried out mainly by speech-language pathologists, singing teachers, and some physicians.
**PRACTICE**	From today to the future, to reach an aim or solve a dilemma, in any aspect of life, personal or professional, in situations that still need coaches.	From present to the future, to reach an aim or solve a dilemma, in the voice or communication area, for professionals who need coaches.
**WORKING PROCESS**	Objectives previously defined by the client (and/or sponsor of the process, in case of organizations), rendering service contract, predetermined start-mid-end time, with the possibility of a third-party sponsor; work is developed between six and 12 months, consisting of 10 to 12 sessions, in average; the same client may hire other process in the future to set different objectives.	Objectives defined along the relationship, together with the client, considering the improvement of performance in the areas of voice and communication, both in the pedagogical, health or artistic point of views; normally, this partnership does not require a contract between the parts and the objectives may be only focal ones, developed in the short term and ending when the result in the performance is reached; this relationship may extend over long time, maybe years, in a continuous or intermittent way, according to the clients request, with a challenge of several voice or communication improvements.
**BASIC KNOWLEDGE**	This professional does no need to have experience or knowledge in this field and client performance areas since she/he acts on the clients‘ thoughts and not on the problem or situation or even coaching dilemmas that are brought into the session.	This professional has pedagogical, rehabilitation or medical experience in the voice or interpersonal communication area, since she/he acts on understanding the clients‘ thoughts, but also guides on the best options for a better performance and voice health.
**PROFESSIONAL PARTNERSHIHP**	Reaching these objectives depends exclusively on the coaching process in the coach-client relationship; eventually the coach may suggest therapy, mostly psychological therapy, and the coach should not assume this position, even if she/he has an academic education for that.	Reaching these objectives may require a joint action with other professionals; eventually the vocal coach may also be a speech-language pathologist, a singing teacher, or the client’s physician, thus being responsible for voice health or pedagogic issues, but also acting as a vocal coach, thus contributing to thinking on the performance, career, and vocal conditioning.
**PROFESSIONAL TITLE**	Professional coach; may be focused on a specialization or specification on the interest area: executive coach, team coach, life coach or career coach; regarding the professionals accredited at ICF, the level of accreditation may be included. *Associate Certified Coach* (ACC)*, Professional Certified Coach* (PCC) and *Master Certified Coach* (MCC)^(3)^; there are other certifications and classifications with respect to other associations or education schools.	We suggest using the academic title of the professional or his/her professional occupation and have vocal coach added as an extra qualification, for example: speech-language pathologist and vocal coach, singing teacher and vocal coach or physician and vocal coach. Thus, the context of the professional performance can be transparent and may support the vocal coach intervention.

## RESULTS

The perceptions and experiences of the six professors are organized in Box 1, with the main similarities and specificities of professional coaching and vocal coaching processes, as well as the respective instructor’s functions and responsibilities, the professional coach and the vocal coach.

## DISCUSSION

Despite the large amount of studies addressing professional coaching^(1-4,8-13)^ in the literature, publications on vocal coaching are rare^(6,7)^.

The official definition proposed by the International Coaching Federation (ICF), the main non-for-profit global organization dedicated to the education and development of coaches, states that “professional coaching is a partnership with the client in a thought-provoking and creative process that inspires them to maximize their personal and professional potential” ^(3)^. ICF recognizes the rapid development of this profession, considered the fastest growing one in the world, especially the executive coaching^(3,8-10)^, as well as the existence of a wide variety of modalities in which coaching approaches and competencies may be applied, proposing the so-called “coaching continuum”. Such a professional continuum includes practicing professional coaches, managers, leaders, human resources executives, and health professionals who apply coaching approaches, strategies, and skills in their workplaces^(3)^. Presently, coaching is not regulated in any country or state, however, there are several coaching schools and associations that offer licensing, continued training, and even post-graduation courses in the area^(3)^.

Coaching objectives, mentioned by several professional education schools, include well-being improvement, personal and professional development, resilience, decision taking, emotional self-regulation, teamwork, and empathy^(2,3)^. The first randomized controlled clinical trial on the use of coaching was developed in Australia^(11)^ about 10 years ago and has shown evident results in 41 executives after a 10-week long program.

The coaching structure is different from training, therapy, consultancy, or mentoring since the process involved requires a differentiated dialog, called coaching conversation. Coaching conversations are a specific kind of communication in which the professional, the coach, concentrates on the clients‘ mind, their thoughts and believes, while the client concentrates on the situation aimed by the coaching process. The coach does not establish actions, does not drive choices nor counsels on specific problems. The professional coach uses powerful questioning to encourage thinking and produce actions. In a single phrase, professional coaching may be defined as “a process to facilitate a positive change by improving thinking in order to unlock a person’s potential to maximize its own performance” ^(12)^. 

In contrast, the terms vocal coaching/coach refer to the performance scenario with singers to help them in rehearsals or performances, and more recently has expanded to help actors, communicators, and speakers^(13)^. More specifically in English-speaking countries, the position of vocal coach is frequently associated with a professional who contributes for improving of specific skills, which may be an opera arias *répétiteur*; a breath, phrasing or pronunciation specialist in several languages, among other skills^(14)^. This description does not mirror the present scenario of the voice area, therefore, we have proposed a different perspective of the concept based on our understanding of vocal coaching/coach^(7)^. A vocal coach should be seen as a professional who aims to improve communication and vocal, artistic, and non-artistic performance, using coaching strategies to reach specific aims related to performance^(7)^. Artistic vocal coaching is mainly proposed for singers, actors, radio speakers, and dubbing artists, while non-artistic vocal coaching is mainly addressed to speakers, executives, politicians, and journalists, among others. The function of vocal coach may be developed by individuals from different academic fields, such as SLP Voice specialists, singing specialists, voice teachers, music professors, artistic directors, theater directors, physicians, and psychologists, among others.

In 2011, the *Centro de Estudos da Voz* (CEV, in English Center for Voice Study) in São Paulo, a private education institution, started offering a one-year long post-graduation program, called Integrated Voice Formation (*Formação Integrada da Voz* – FIV), which was the base to suggest, in 2018, a FIV focused on coaching – FIV-C. It was offered with an average of 300 hours of duration with one of the goals being to offer a common understanding to the several professionals acting in the voice area, such as speech-language pathologists, singing teachers, physicians, among others, and provide them with skills like the knowledge of professional coaching strategies^(15)^. The FIV-C program suggested using the term “vocal coach” as a supplementary qualification to the original academic graduation or professional position, for example: speech-language pathologist voice specialist, and vocal coach, singing teacher and vocal coach, or even otolaryngologist and vocal coach.

The analysis performed and the chart proposed with the main similarities and differences contribute to make clear that professional coaching is a comprising process, although the existence of several specializations and contexts of this service rendering are recognized. Persons from the most varied academic fields, such as Administration, Engineering, Economy, Psychology, and other health professions aim at a formation accredited in coaching to act as professional coaches. The professional coach does not require some knowledge on the topics related to a coaching process, which are raised by the client. The vocal coach, however, has a specific practice and, although presently a special certification may not be required to identify the professional as such, we consider that a post-graduation that offers training in models, strategies, and dynamics on coaching should be recommended. Opposite to the professional coach, a vocal coach requires some experience in the voice area, a designation used by speech-language pathologists, singing or oratory teachers and doctors. The vocal coach function may be employed along with the original academic education, both in the health area and vocal pedagogy. It is important to highlight that identifying oneself as vocal coach is a mere consequence of having a short designation, although it is recognized that the most adequate name should be communication and vocal coach, since voice is considered the base on which oral communication is built. Thus, improving communicative competence is the final aim of vocal coaching. The next decade will potentially bring the possibility to consolidate a position of vocal coach, since professionals who perform this function have greater visibility in the market by announcing its results in scientific events as well as producing research and papers in specialized journals.

## CONCLUSION

Professional coaching and vocal coaching are human development processes, the former is more general and the latter is more focused on vocal performance, but both target moving from the present to the future situation by seeking to solve dilemmas and reach objectives. The main differences rely on the need for a specific training to act as professional coach, the pre-requisite of formulating a contract and predefined objectives. The vocal coach, conversely, requires neither specific training nor a contract for service rendering; in addition, the goals may be set by consulting with the client. Professionals from several academic graduations may certify in professional coaching; however, vocal coaching is commonly practiced by speech-language pathologists, singing teachers, and physicians. We suggested that the designation of vocal coach should be added to the original academic education or professional practice title.

## References

[1] O’Connor J, Lages A (2007). How coaching words: the essential guide to the history and practice of effective coaching..

[2] Bresser F, Wilson C, Passmore J (2016). Excellence in coaching: the industry guide..

[3] ICF: International Coach Federation (2016). Global Coaching Study - executive summary..

[4] Grover S, Furnham A (2016). Coaching as a developmental intervention in organizations: a systematic review of its effectiveness and the mechanisms underlying it. PLoS One.

[5] Lovell B (2018). What do we know about coaching in medical education? A literature review. Med Educ.

[6] Gill BP, Herbst CT (2016). Voice pedagogy - what do we need*?*. Logoped Phoniatr Vocol.

[7] Behlau M, Madazio G, Pacheco C, Vaiano T, Badaró F, Barbara M (2021). Coaching strategies for behavioral voice therapy and training. J Voice.

[8] ICF: International Coach Federation (2020). 2020 ICF Global Coaching Survey - executive summary.

[9] Sperry L, Sperry L (2002). Effective Leadership: Strategies for Maximizing Executive Productivity and health..

[10] ICF: International Coach Federation Global consumer awareness study.

[11] Grant AM, Curtayne L, Burton G (2009). Executive coaching enhances goal attainment, resilience and workplace well-being: a randomised controlled study. J Posit Psychol.

[12] Rock D (2007). Quiet leadership: six steps to transforming performance at work..

[13] Oxford Dictionary (2020). Vocal coach.

[14] Definitions (2020). Vocal coach.

[15] CEV: Centro de Estudos da Voz (2020). Formação Integrada em Voz com foco em Coaching – FIVC.

