# Supplementary material for: Professional coaching versus vocal coaching: similarities and differences
Source: Codas. 2022 Feb 23;34(4):e20210003. doi: 10.1590/2317-1782/20212021003 (PMC9886100; doi:10.1590/2317-1782/20212021003)
Supplement: Translation

# Translation: Coaching profissional versus coaching vocal: similaridades e diferenças

**Authors:** Mara Behlau, Glaucya Madazio, Claudia Pacheco, Thays Vaiano, Flávia Badaró, Marisa Barbara

**Affiliations:** 1 Centro de Estudos da Voz – CEV, São Paulo (SP), Brasil.; 2 Universidade Federal de São Paulo – UNIFESP, São Paulo (SP), Brasil.; 3 Insper, São Paulo (SP), Brasil.

**Keywords:** Coaching, Treinamento, Performance, Voz, Comunicação

## Abstract

**Objetivo:**

essa Comunicação Breve descreve coaching/coach profissional e faz um contraponto com o coaching/coach vocal. O objetivo desse artigo é trazer clareza nessas duas apresentações de coaching, contribuindo para o uso correto e diferencial dos termos em questão.

**Método:**

seis professores de um curso de pós-graduação, fonoaudiólogos e/ou coaches profissionais e/ou coaches vocais reuniram-se e compartilharam suas percepções e experiências em coaching profissional ou vocal e no ensino de estratégias de coaching para profissionais que atuam na área da voz. Um quadro de semelhanças e diferenças entre as duas atribuições, tanto quando ao processo de intervenção como quanto à formação profissional.

**Resultados:**

seis aspectos fundamentais foram identificados para caracterizar as apresentações de coaching profissional e vocal: credencial, atuação, processo, conhecimento básico, parceria para resultados e título profissional.

**Conclusão:**

o coaching profissional é um processo estruturado que exige formação certificada, com indivíduos credenciados por associações qualificadas e tem como objetivo facilitar mudanças positivas na vida dos clientes, melhorando a qualidade do pensamento para desenvolvimento de habilidades pessoais e profissionais. Já o coaching vocal é uma função não estruturada, que não exige formação certificada, podendo ser desenvolvida por diversos profissionais da saúde ou pedagogia vocal, com o objetivo de melhorar a performance vocal e/ou comunicativa de indivíduos usuários de voz artística ou não artística, cantada ou falada. Particularmente, quanto ao coaching vocal, sugere-se a manutenção da identificação profissional pela formação acadêmica ou atuação profissional de base, acrescentando-se o termo coach vocal, como qualificador.

## INTRODUÇÃO

A origem da palavra *coaching* não é bem definida, mas há registros de que ela possa ter aparecido, pela primeira vez, no século 16 no húngaro medieval, como *kocsi*, em referência às famosas “carruagens de Kocs”, uma vila na qual esses luxuosos meios de transporte eram construídos. De *kocsi* para *coche* no francês médio e *kotsche*, no alemão, o verbete aparece mais tarde na Inglaterra, na primeira metade do século 19 como *coach*, para indicar um professor particular que “conduz” um aluno pelo processo de se submeter a um exame. Na segunda metade desse mesmo século, tanto na Inglaterra quanto nos Estados Unidos, a palavra *coach* passou a ser utilizada para se referir especificamente a um instrutor ou treinador esportivo, no sentido atlético^(1)^. Portanto, o significado subjacente a *coach* é o de alguém que acompanha um indivíduo em uma jornada sofisticada e contribui para que se chegue em um determinado destino. Do cenário esportivo, o *coaching* foi transferido para o mundo das organizações nas décadas de 1970 e 1980, gerando um enorme grau de diversificação na década de 1990, profissionalizando-se e popularizando-se no presente século, no qual se consolidou como uma das profissões que mais cresce no mundo^(2)^.

Desta forma, as palavras *coaching* e *coach* têm sido cada vez mais usadas em textos leigos e na literatura científica, mantendo-se sua forma em inglês, sem tradução equivalente para o português. Entende-se por *coaching* o processo e por *coach* o profissional habilitado para essa atividade específica. Não são claras as diferenças entre esse procedimento e outras modalidades de atendimento, e também entre o profissional responsável por ele e outros prestadores de serviço, tais como professores, clínicos e treinadores. De modo geral, o *coaching* refere-se a uma abordagem de desenvolvimento humano, mas, para contribuir na compreensão do que esse processo e essa função significam, frequentemente acrescem-se qualificadores, indicando o contexto em que esse termo está sendo utilizado, tais como: *coaching/coach* profissional, executivo, carreira, sistêmico, Ericksoniano, de vida, de esportes e vocal. Quem conduz o processo é chamado de *coach* e quem contrata esse serviço era, inicialmente, designado *coachee;* contudo, ultimamente, prefere-se usar o termo cliente, de mais fácil pronúncia e compreensão^(3)^.

A literatura sobre *coaching* profissional e sua contribuição nas organizações é extensa e os resultados mostram efeitos na produtividade e *performance* de executivos, com nível de evidência variável^(4)^. Também na educação médica ele é apresentado como uma modalidade de treinamento, com elevada evidência que apoia o uso desse processo para a melhoria das habilidades técnicas^(5)^.

Na Fonoaudiologia, os especialistas em voz têm utilizado esses dois termos, *coaching* e *coach* vocal, de modo mais presente nas mídias sociais. Consideramos importante trazer maior clareza ao que significam. A literatura sobre *coaching* vocal é bastante limitada e destaca-se um artigo específico^(6)^ que apresenta um resumo de um painel de professores de canto durante a 10ª *Pan-European Voice Conference* - PEVOC, no qual são propostas três áreas na pedagogia da voz: *voice building*, *coaching* vocal e reabilitação da voz. Nele, o *coaching* vocal é apresentado como a capacidade de transferir e estabelecer habilidades que estão diretamente relacionadas com a *performance* em palco ou estúdio e a expressividade artística. O principal requisito para desempenhar essa função de *coach* vocal é ter tido experiência profissional ativa e/ou de apoio em atuação vocal ou em gravações em estúdio. Tal proposta é um marco na área, embora não tenha sido explorada com profundidade. Recentemente, um tutorial apresentando o uso de estratégias de *coaching* no treinamento de voz e na reabilitação de indivíduos com disfonias comportamentais oferece um panorama dos recursos dessa modalidade de interação que podem ser usados na área da saúde e na pedagogia vocal^(7)^.

O objetivo do presente artigo é o de compilar as principais similaridades e diferenças entre os chamados *coaching* profissional e *coaching* vocal, no sentido de contribuir para que haja clareza entre os processos relacionados a essas duas situações e às funções profissionais desenvolvidas pelos indivíduos que ministram esse tipo de atendimento.

## MÉTODO

Esse artigo é o resultado de uma análise reflexiva dos autores, professores de pós-graduação; a investigação não utilizou dados de sujeitos e, desta forma, entendemos que não seja necessária aprovação de Comitê de Ética e nem TCLE.

Seis professores de um curso de pós-graduação em voz, nível aperfeiçoamento, reuniram-se para compartilhar suas percepções e experiências sobre os processos de *coaching* profissional e *coaching* vocal e sobre a atuação do *coach* profissional e do *coach* vocal. Foram selecionados seis aspectos fundamentais essenciais, com o objetivo de contribuir na caracterização dessas duas apresentações de *coaching*, a saber: 1. Formação: necessidade de credencial para o exercício profissional e níveis de certificação; 2. Atuação: direção da atitude profissional frente ao cliente; 3. Processo de trabalho: definição de objetivos, contrato e duração dos atendimentos; 4. Conhecimento básico na área de demanda do cliente: necessidade de formação nos temas trazidos pelo cliente; 5. Parceria para resultados: caracterização da relação entre o *coach* e o cliente, com indicação da responsabilização pelos resultados; e, finalmente, 6. Título profissional: identificação da qualificação do *coach*. Os resultados dessa discussão, ocorrida em três encontros de uma hora cada, são apresentados no Quadro 1.

**Quadro 1:** Principais similaridades e diferenças entre *coaching* e *coach* profissional e vocal

**ASPECTOS**	***COACHING* e *COACH* PROFISSIONAL**	***COACHING* e *COACH* VOCAL**
**FORMAÇÃO**	Titulação em Escola de *Coaching* Profissional (acreditada pela ICF ou outra instituição certificadora); a ICF credencia *coaches* em três níveis: ACC, PCC e MCC; possui um código de ética e 8 competências a serem respeitadas^(3)^; profissionais das mais variadas formações acadêmicas buscam certificação para atuarem como *coach* profissional.	Não requer titulação e qualquer pessoa, sem formação específica, pode-se intitular *coach* vocal; o mercado reconhece o valor na sua especialidade; não há níveis de certificação; espera-se obediência ao código de ética de sua profissão e não há descrição de competências; a função de *coach* vocal é exercida principalmente por fonoaudiólogos, professores de canto e alguns médicos.
**ATUAÇÃO**	Movimento de hoje para o futuro, para atingir um objetivo ou resolver um dilema, em quaisquer áreas da vida, pessoal ou profissional, em situações que sejam motivos de *coaching.*	Movimento de hoje para o futuro, para atingir um objetivo ou resolver um dilema, na área de voz ou de comunicação, em situações profissionais, que sejam motivos de *coaching.*
**PROCESSO DE TRABALHO**	Objetivos pré-definidos pelo cliente (e/ou patrocinador do processo, no caso de organizações), contrato de prestação de serviços, tempo pré-determinado para início-meio-fim, com possibilidade de um patrocinador terceiro; o trabalho é desenvolvido entre 6 e 12 meses, com 10 a 12 sessões, em média; o mesmo cliente pode contratar outro processo no futuro para trabalhar outros objetivos.	Objetivos definidos ao longo da relação, em conjunto com o cliente, considerando-se melhora de *performance* na área de voz e de comunicação, seja do ponto de vista pedagógico, de saúde ou artístico; geralmente, essa parceria não exige contrato entre as partes e os objetivos podem ser apenas focais, desenvolvidos no curto-prazo, finalizando-se quando o resultado na *performance* tenha sido alcançado; essa relação pode-se estender por um período maior, ao longo de anos, de forma contínua ou intermitente, de acordo com a solicitação do cliente, com desafios de desenvolvimento vocal ou de comunicação diversos.
**CONHECIMENTO BÁSICO**	Esse profissional não precisa ter nenhuma experiência ou conhecimento na área de interesse e atuação do cliente, pois atua sobre o pensamento do cliente e não sobre o problema, a situação ou os dilemas de *coaching* trazidos para as sessões.	Esse profissional tem experiência pedagógica, de reabilitação ou médica na área de voz ou comunicação interpessoal, pois atua sobre o pensamento do cliente, mas também orienta sobre as melhores opções para a *performance* e saúde vocal.
**PARCERIA PROFISSIONAL**	O alcance dos objetivos depende exclusivamente do processo de coaching na relação *coach*-cliente; eventualmente o *coach* pode sugerir terapia, geralmente psicológica, não devendo o *coach* ocupar essa segunda posição, mesmo que tenha formação acadêmica para tanto.	O alcance dos objetivos pode exigir a ação conjunta de outros profissionais; eventualmente o *coach* vocal pode também ser o fonoaudiólogo, professor de canto ou médico do cliente, sendo responsável por questões de saúde vocal ou pedagógica, mas também atuando como *coach* vocal, contribuindo para reflexões sobre *performance*, carreira e condicionamento vocal.
**TÍTULO PROFISSIONAL**	*Coach* profissional; pode-se acrescentar uma especialização ou especificação da área de interesse: *coach* executivo, *coach* de time, *coach* de vida ou *coach* de carreira; no caso de profissionais registrados na ICF pode-se acrescentar o nível de credenciamento: *Associate Certified Coach – ACC, Professional Certified Coach – PCC e Master Certified Coach – MCC* ^(3)^; há outras certificações e classificações em outras associações ou escolas de formação.	Sugerimos manter o título da formação acadêmica do indivíduo ou de sua ocupação profissional e acrescentar *coach* vocal como uma qualificação adicional; por exemplo: fonoaudiólogo e *coach* vocal, professor de canto e *coach* vocal ou médico e *coach* vocal; desta forma, o contexto da atuação profissional fica mais claro e pode servir de base para a intervenção do *coach* vocal.

## RESULTADOS

O compartilhamento das percepções e experiências dos seis professores foi organizado no Quadro 1, que apresenta as principais similaridades e particularidades dos processos de *coaching* profissional e *coaching* vocal, assim como das funções e responsabilidades do ministrante desse processo, o *coach* profissional e o *coach* vocal.

## DISCUSSÃO

Encontra-se na literatura uma grande quantidade de material sobre o *coaching* profissional^(1-4,8-13)^, mas há raras publicações sobre o *coaching* vocal^(6,7)^.

A definição oficial proposta pela *International Coaching Federation* (ICF), a principal organização global sem fins lucrativos dedicada à formação e desenvolvimento de *coaches,* diz que “*coaching* profissional é uma parceria com clientes em um processo criativo e instigante que os inspira a maximizar seu potencial pessoal e profissional"^(3)^. A ICF reconhece o franco desenvolvimento dessa profissão, considerada a que mais cresce no mundo, principalmente o *coaching* executivo^(3,8-10)^ e, também, a existência de uma gama mais ampla de modalidades nas quais as abordagens e competências de *coaching* podem ser aplicadas, propondo o chamado “*continuum* de *coaching*”. Nesse *continuum* profissional estão incluídos *coaches* profissionais praticantes, gerentes, líderes, executivos de recursos humanos e profissionais de saúde que aplicam abordagens, estratégias e habilidades de *coaching* em seu local de trabalho^3^. Atualmente, o *coaching* não é regulamentado por nenhum país ou estado, porém, existem diversas escolas e associações de *coaches* que oferecem credenciamento, treinamento continuado e até mesmo pós-graduações na área^3^.

Os objetivos do *coaching*, mencionados por diversas escolas de formação profissional, incluem melhoria de bem-estar, desenvolvimento pessoal e profissional, resiliência, tomada de decisão, autorregulação emocional, trabalho em equipe e empatia^(2,3)^. O primeiro estudo clínico controlado randomizado sobre o uso do *coaching* foi desenvolvido na Austrália^(11)^ há 10 anos e mostrou resultados evidentes em 41 executivos após um programa de 10 semanas.

A estrutura de *coaching* é diferente de treinamento, terapia, consultoria ou mentoria. O processo de *coaching* requer um diálogo diferenciado, denominado conversas de *coaching*. As conversas de *coaching* são um tipo de comunicação específica na qual o profissional, o *coach*, concentra-se na mente do cliente, em seu pensamento e suas crenças, enquanto o cliente concentra-se na situação que é motivo de *coaching*. O *coach* não determina ações, não dirige escolhas e nem aconselha sobre determinados problemas. O *coach* profissional usa perguntas poderosas para estimular a reflexão e produzir ações. Em uma simples frase, o *coaching* profissional pode ser definido como "um processo para facilitar a mudança positiva, melhorando o pensamento, a fim de desbloquear o potencial de uma pessoa para maximizar seu próprio desempenho”^(12)^.

Por sua vez, os termos *coaching/coach* vocal referem-se ao cenário de atuação com cantores, para auxiliá-los em ensaios ou apresentações, expandindo-se mais recentemente para auxiliar atores, comunicadores e palestrantes^(13)^. Mais especificamente em países de língua inglesa, a função de *coach* vocal aparece frequentemente associada a um profissional que contribui para a melhoria de habilidades específicas, podendo ser um repassador de árias de ópera, um especialista em respiração, fraseado, pronúncia em diversas línguas, dicção e outras habilidades^(14)^. Essa descrição não espelha a situação atual que observamos na área de voz e, recentemente, propusemos uma diferente perspectiva de conceito do que entendemos sobre *coaching/coach* vocal^(7)^. O *coach* vocal deve ser visto como um profissional que visa aprimorar o desempenho comunicativo e vocal, artístico e não artístico, utilizando estratégias de *coaching* profissional para atingir objetivos específicos relacionados à *performance* ^(7)^. O *coaching* vocal artístico é geralmente proposto para cantores, atores, locutores e dubladores enquanto o *coaching* vocal não artístico é geralmente endereçado a palestrantes, executivos, políticos e repórteres, entre outros. A função de *coach* vocal pode ser desenvolvida por indivíduos de diferentes formações acadêmicas, como fonoaudiólogos, especialistas em canto, professores de voz, professores de música, diretores artísticos, diretores de teatro, médicos e psicólogos, entre outros.

Em 2011, o Centro de Estudos da Voz - CEV, em São Paulo, instituição de ensino privada, passou a oferecer um programa de pós-graduação de um ano, denominado Formação Integrada em Voz - FIV, que foi a base para se propor, em 2018, o FIV com foco em *coaching* – FIV-C, com uma média de 300 horas de duração tendo como um de seus objetivos oferecer uma linguagem comum aos diversos profissionais que atuam na área de voz: fonoaudiólogos, professores de canto, médicos, entre outros, e instrumentalizá-los com o domínio de estratégias de *coaching* profissional^(15)^. O programa FIV-C sugere o uso do termo “*coach* vocal” como uma qualificação complementar à formação acadêmica ou função profissional de origem, por exemplo: fonoaudiólogo especialista em voz e *coach* vocal, professor de canto e *coach* vocal, ou ainda médico otorrinolaringologista e *coach* vocal.

A reflexão realizada e o quadro proposto com as principais similaridades e diferenças contribuem para esclarecer que o *coaching* profissional é um processo mais abrangente, embora se reconheça a existência de diversas especializações e contextos da prestação de serviço. Indivíduos das mais variadas formações acadêmicas, tais como administradores, engenheiros, economistas, psicólogos e outros profissionais da saúde, buscam uma formação acreditada em *coaching* para atuar como *coach* profissional. O *coach* profissional não precisa ter nenhum conhecimento sobre os tópicos passíveis de um processo de *coaching*, trazidos pelo cliente. Já o *coach* vocal tem uma atuação mais específica e embora, no presente momento, não seja exigida nenhuma certificação especial para identificar-se como tal, entendemos que seja recomendável uma formação de pós-graduação que ofereça treinamento de modelos, estratégias e dinâmicas da disciplina de *coaching.* Ao contrário do *coach* profissional, para o exercício do *coach* vocal deve-se ter experiência na área de voz e essa designação é utilizada por fonoaudiólogos, professores de canto ou de oratória e médicos. A função de *coach* vocal pode ser alternada com a da formação acadêmica original, seja na área da saúde ou da pedagogia vocal. Uma observação a ser feita é a de que identificar-se como *coach* vocal é apenas uma consequência de se ter um nome curto, embora se reconheça que a designação mais adequada deveria ser *coach* vocal e de comunicação, já que a voz é vista como o alicerce sobre o qual a comunicação oral é construída, sendo a melhoria da competência comunicativa o objetivo ulterior ao do *coaching* vocal. A próxima década tem potencialmente a possibilidade de consolidar a função de *coach* vocal, na medida em que os profissionais que exercem essa função passem a adquirir maior visibilidade no mercado, divulgando seus resultados em eventos científicos e produzindo pesquisas e artigos em revistas especializadas.

## CONCLUSÃO

O *coaching* profissional e o *coaching* vocal são processos de desenvolvimento humano, o primeiro mais geral e o segundo mais focado na performance vocal, ambos com um movimento de hoje para o futuro, na solução de dilemas e conquistas de objetivos. As principais diferenças residem na necessidade de formação específica para atuar como *coach* profissional, o pré-requisito da formulação de um contrato e objetivos pré-determinados; já o *coach* vocal não exige uma formação específica, nem contrato de prestação de serviço e os objetivos podem ser determinados nos atendimentos ao cliente. Profissionais de diversas formações acadêmicas podem certificar-se no *coaching* profissional; já o *coaching* vocal é comumente exercido por fonoaudiólogos, professores de canto e médicos. Sugere-se que a designação de *coach* vocal seja acrescida à formação acadêmica ou atuação profissional original.
